# Interaction of Iron Homeostasis and Fatty Acid Metabolism in the Development of Glucose Intolerance in Women with Previous Gestational Diabetes Mellitus

**DOI:** 10.3390/nu15143214

**Published:** 2023-07-20

**Authors:** Kristin Källner, Rasmus Krook, Ann-Sofie Sandberg, Lena Hulthén, Ulrika Andersson-Hall, Agneta Holmäng

**Affiliations:** 1 Institute of Neuroscience and Physiology, Sahlgrenska Academy, University of Gothenburg, 405 30 Gothenburg, Swedenulrika.andersson.hall@gu.se (U.A.-H.);; 2 Division of Food and Nutrition Science, Department of Life Sciences, Chalmers University of Technology, 412 96 Gothenburg, Sweden; 3 Institute of Medicine, Sahlgrenska Academy, University of Gothenburg, 413 45 Gothenburg, Sweden

**Keywords:** gestational diabetes mellitus, glucose tolerance, insulin resistance, iron, transferrin receptor, serum fatty acids

## Abstract

A gestational diabetes mellitus (GDM) diagnosis during pregnancy means an increased risk of developing type 2 diabetes later in life. By following up with women after GDM we aimed to examine the relationship between iron parameters, individual fatty acids (FAs) and desaturases in the development of impaired glucose metabolism (IGM). Based on an oral glucose tolerance test (OGTT), six years after GDM, 157 women were grouped as having normal glucose tolerance (NGT) or IGM. Fasting serum FAs, activity of desaturases and iron parameters (ferritin, transferrin, iron, soluble transferrin receptor, total iron binding capacity, hepcidin) were measured, and clinical and anthropometric measurements taken. Soluble transferrin receptor was higher in the IGM group compared to the NGT group (3.87 vs. 3.29 mg/L, *p*-value = 0.023) and associated positively with saturated FAs and negatively with monounsaturated FAs in the IGM group (adjusted for BMI, age and high sensitivity C-reactive protein; *p*-value < 0.05). Iron, as well as transferrin saturation, showed a positive association with MUFAs and desaturase activity. These associations were not seen in the NGT group. These results suggest that iron homeostasis and FA metabolism interact in the development of glucose intolerance in women with previous GDM.

## 1. Introduction

Gestational diabetes mellitus (GDM) is defined as the onset or first recognition of any degree of glucose intolerance during pregnancy [1]. GDM and obesity increases the risks of pregnancy complications and increases the risks for the child (high-birth weight, cesarean section, neonatal hypoglycemia, high cord-blood serum C peptide, premature delivery, shoulder dystocia, intensive neonatal care, hyperbilirubinemia, preeclampsia, accelerated growth patterns, foetal growth restriction) [2,3]. The elevated glucose levels generally normalize immediately postpartum. However, GDM can expose an existing underlying deficiency in insulin secretion and sensitivity, revealing a predisposition to develop type 2 diabetes (T2D) [4,5]. Women with GDM have an almost ten times higher risk of developing T2D than women who have had a normoglycemic pregnancy, and the risk is highest during the first five years after pregnancy [6].

Fatty acids (FAs), acquired through diet or synthesis, are believed to have substantial impact on the development of T2D and insulin resistance by various mechanisms. Elevated circulating saturated FAs (SFAs) contribute to lipotoxicity and lipoapoptosis, processes that lead to pancreatic β-cell dysfunction and apoptosis [7]. Several studies suggest that SFAs induce, while unsaturated FAs inhibit, β-cell death [8]. The plasma fatty acid profile is influenced not only by dietary fat intake, but also by endogenous fatty acid metabolism, e.g., by desaturase enzymes. These enzymes insert double bonds between carbon atoms of the fatty acid chain and thereby regulate the degree of lipid unsaturation throughout the body and may impact factors involved in the development of glucose intolerance [9]. Glucose and lipid metabolism disorders can be the result of abnormal bile acid levels. Women with intrahepatic cholestasis of pregnancy are more likely to develop GDM, and GDM independently contributes to adverse pregnancy outcomes among women with intrahepatic cholestasis of pregnancy. However, the combined effects of GDM and maximum total bile acid concentration on adverse pregnancy outcomes do not appear to be multiplicative or additive [10]. We previously reported that the fatty acid profile in women, as well as the activity of desaturases differed depending on glucose tolerance 6 years after GDM [11].

In recent decades, accumulating evidence has shown that iron is critical for maintaining body homeostasis and is an important factor in the development of glucose intolerance and T2D through several mechanisms. Iron is potentially toxic as it generates oxidative stress by catalysing the generation of reactive oxygen species (ROS), leading to β-cell damage and dysfunction. Iron also affects hypoxia inducible factors, which can downregulate glucose transporters and decrease glucose tolerance [12]. In an experiment with stressed rats, a high-iron diet induced persistent hyperglycemia, and aggravated stress induced iron deposition and oxidative stress injury to the liver [13]. It has also been shown that iron metabolism is related to insulin resistance in GDM women, suggesting that change in iron metabolism may be involved in the pathogenesis of GDM by aggravating stress hyperglycemia [14]. Compared to other blood constituents, serum ferritin has a very wide “normal” range and it has been suggested that this broad range may include levels that associate with health risks yet to be identified. It is evident that there is a strong relationship between ferritin and T2D risk, even across the normal range of ferritin [15]. Recent genetic evidence supports a causal link between increased systemic iron status and increased T2D risk [16].

Iron interacts with lipid metabolism directly and indirectly. The ways in which these interact are numerous and not fully understood. A study in rat models showed that iron overload affected serum levels of glucose, insulin and insulin resistance differently, depending on the level of fatty acid saturation of the diet [17]. Rat studies have also shown interaction between iron levels and desaturases, where high iron levels are associated with both lower delta-5 desaturase (D5D) activity and delta-6 desaturase (D6D) (inserting double bonds after the 5th resp. 6th carbon atom) activity [18], as well as increasing stearoyl-CoA desaturase (SCD) activity [19]. A new form of regulated cell death referred to as ferroptosis is an iron dependent process that can contribute to the loss of β-cells subjected to lipid peroxidation [20]. Recently a study presented differential effects of saturated and unsaturated FAs on ferroptosis in rat β-cells [21]. In summary, iron, FAs, desaturases, and their interactions seems to affect glucose tolerance, but to our knowledge no studies in human subjects investigating these associations exist. Our aim was to examine the relationship between iron parameters, individual serum fatty acids and desaturases in a population previously diagnosed with GDM and with a higher risk of developing T2D. We wanted to explore if and how these differed between women who had maintained normal glucose tolerance (NGT) and those with impaired glucose metabolism (IGM) to better understand the underlying pathogenesis and search for early biomarkers in the development of insulin resistance.

## 2. Materials and Methods

### 2.1. Subjects

**Figure 1:**
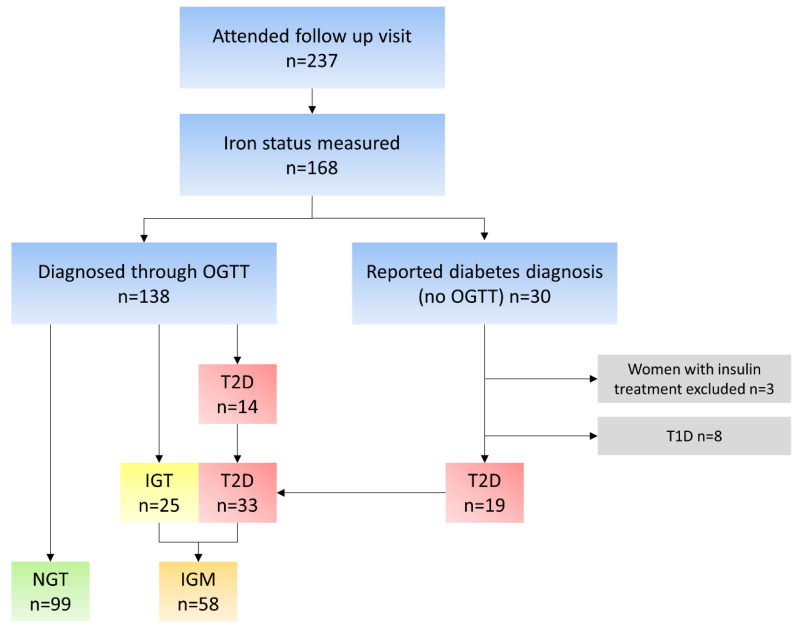
Flow chart of the study population and reason for exclusion. n, number; OGTT, oral glucose tolerance test; T2D, type 2 diabetes; T1D, type 1 diabetes; IGT, impaired glucose tolerance; NGT, normal glucose tolerance; IGM, impaired glucose metabolism.

All women in the Gothenburg area who were diagnosed with GDM from 29 December 2004 to 26 September 2007 (n = 291) were eligible to participate in the study; 241 of those women were interviewed by telephone and asked to attend a follow-up visit; 169 women attended the follow-up visit 5.6 ± 0.5 years after pregnancy, between 1 April 2011 and 28 February 2014. Excluded from the study were eight women that had been diagnosed with T1D and three more that were being treated with insulin. One was excluded due to an error in the handling of blood samples. The remaining 157 women comprise the study population (Figure 1).

This study was approved by the ethical committee at the University of Gothenburg (dnr 402-08/T232-11). Written consent was received from all participants.

### 2.2. Collection of Data and Data Processing

Capillary fasting glucose was collected to examine if the participants could proceed with an oral glucose tolerance test (OGTT). All women with adequately low capillary fasting glucose (<8 mmol/L) that had not previously received a diabetes diagnosis underwent a two-hour 75 g OGTT. Venous blood for analysis of plasma glucose and serum insulin was collected at 0 (fasting value), 30, 60, 90, and 120 min. Capillary glucose was also collected at 120 min in case of unsuccessful venous blood sampling. Fasting serum and plasma blood samples were used for further analysis of iron parameters, high sensitivity C-reactive protein (hs-CRP), fatty acids and desaturase activity measured as the ratio between fatty acid converted and fatty acid produced [11]. Anthropometric measurements including height, waist circumference, hip circumference and weight were also determined at the visit.

### 2.3. Glucose Tolerance Groups

The women were assigned two different groups depending on venous glucose values from the OGTT at 0 and 120 min (Figure 1), according to 2006 WHO guidelines [22]. The first group were women with normal glucose tolerance (NGT); i.e., glucose levels <6.1 at 0 min and <7.8 at 120 min. The second group, impaired glucose metabolism (IGM), was comprised of women with impaired glucose tolerance, impaired fasting glucose or T2D, which also included women previously diagnosed with T2D. These women had a glucose level >6.1 at 0 min and/or >7.8 at 120 min. These glucose levels were only used for classification and the values were not used in further analyses.

### 2.4. Biochemical Measurements

Glucose, insulin, ferritin, iron, transferrin, soluble transferrin receptor (sTfR), transferrin saturation (TSAT), TIBC and hs-CRP were analysed, in conjunction with sample collection, at the accredited Clinical Chemistry Laboratory, Sahlgrenska University Hospital (International Standard ISO 15189:2007). The coefficient of variation (CV), reporting limit (RL) and reference interval (RI), was used for p-glucose, CV 3%, RL 0.28 mmol/L, RI 4.0–6.0 mml/L (fasting); s-insulin, CV 10%, RL 1.0 mIE/L, RI 2.7–17 mIE/L (fasting); s-ferritin, CV 7%, RL 1 µg/L, RI 15–150 µg/L (women); s-iron, CV 5%, RL 1 µmol/L, RI 9–34 µmol/L; s-transferrin, CV 4%, RL 0.1 g/L, RI 1.9–3.3 g/L; s-sTfR, CV 5%, RL 0.5 mg/L, RI 1.9–4.4 mg/L (women); s-hs-CRP, CV 7%, RI < 5 mg/L. TIBC was calculated as transferrin×25.1 (RI 47–80 µmol/L) and TSAT was calculated as s-iron/s-TIBC. Serum hepcidin was analysed using commercial peptide enzyme immunoassay (EIA), as previously described [23]. The individual serum FA concentration and desaturase activity was determined as fatty acid methyl esters (FAME) using GC mass spectrometry, as previously described [11]. These analyses were performed at Chalmers University of Technology, Food and Nutrition Science Division. For precision and stability, the monitoring of quality control samples was employed. These samples were used for an extended time, both in and between analytical batches. In each batch, additional standards were run and analysed randomly. A CV value of maximum 0.99% was accepted for these standards. For the FA analyses, the maximum CV was 5%. Serum FA composition was calculated as percentages of total FAs. Homeostatic model assessment of insulin resistance (HOMA-IR) was calculated as (fasting glucose × fasting insulin)/22.5. Hepcidin, FA analyses were made directly after completion of blood sample collection.

### 2.5. Statistical Analyses

Statistical analyses were performed using IBM SPSS version 28.0, (Armonk, NY, USA: IBM Corp.). Linear regressions were analysed unadjusted and adjusted for BMI, age and hs-CRP. Normality in both groups (NGT and IGM) was tested with Shapiro–Wilk and by inspecting the spread of data in histograms. Dependent variables in linear regressions were all normally distributed. Groups were compared with Mann–Whitney U. *p* < 0.05 was considered significant.

## 3. Results

### 3.1. Background Characteristics

The study population consisted of 157 women attending a 6 years follow up after GDM: 99 women with NGT and 58 women with IGM. Drop-out analysis of the 157 women who attended the follow-up visit, compared to the 291 women eligible to participate, showed no significant difference in age. However, the plasma glucose level as well as BMI (at start of gestation) was significantly higher in women who did not participate in the study.

As expected, glucose, insulin and HOMA-IR were higher in the IGM group compared to the women with NGT (Table 1). Also, BMI, waist circumference, hip circumference and hs-CRP were significantly higher in the women with IGM in comparison with the NGT group.

### 3.2. Iron Status

Based on iron status measurements (Table 2), the groups differed in iron, sTfR abundance and TSAT. sTfR was higher while iron and TSAT were lower in women with IGM. Ferritin, transferrin, TIBC and hepcidin measurements showed no significant differences between the two groups.

### 3.3. Fatty Acid Profiles 

Levels of fatty acids and desaturase activity have previously been published within the same study [11]. Results for the current subpopulation are presented in Table 3, grouped by saturation status. Palmitic acid and myristic acid among SFAs were lower in normoglycemic women unlike stearic acid, for which there was no significant difference between NGT and IGM women. There were no differences between the groups regarding monounsaturated fatty acids (MUFAs). Docosapentaenoic acid (DPA) was higher in the IGM group; it was the only polyunsaturated ω-3 fatty acid (ω-3 PUFA) with a significant difference between the groups. Among the ω-6 PUFAs, linoleic acid was lower in the IGM group while γ-linolenic acid and dihomo-γ-linolenic acid were higher in the IGM group compared with the NGT group. Among the desaturases, only Delta-6 activity differed significantly, being higher in the IGM group.

### 3.4. Associations between Fas, Desaturase Activity and Iron Parameters

Correlations between iron status and FAs, as well as desaturase activity, were adjusted for BMI, age and hs-CRP in the linear regression analysis. Table 4 shows all significant associations for the NGT group in the unadjusted or the adjusted model. For a complete list of linear regression analyses for the NGT group, see Table S1. In the NGT group, several significant correlations were found between ω-3 PUFAs and iron status. Ferritin showed a positive correlation with DPA in the unadjusted and adjusted model. Iron, as well as TSAT, correlated positively with docosahexaenoic acid (DHA) but ceased to correlate significantly after adjusting for BMI, age and hs-CRP. Hepcidin showed inverse correlations with the MUFA oleic acid and D6D activity, both unadjusted as well as adjusted. There were no significant associations between iron measurements and SFAs in this group.

Unlike the NGT group, within the IGM group iron parameters that were associated with SFAs, MUFAs and the desaturases converted SFAs to MUFAs. Table 5 shows all significant associations for the IGM group in either the unadjusted or adjusted model. For a complete overview of linear regression analyses in the IGM group, see Table S2.

While serum iron showed a positive association with all three MUFAs analysed, sTfR associated negatively with vaccenic acid and oleic acid. Iron also associated positively with SCD and D9D activity. sTfR also associated positively with the SFAs myristic acid and palmitic acid. Similar to iron, TSAT associated positively with SCD and D9D activity and with the MUFAs, except for palmitoleic acid. TIBC was associated with the SFA stearic acid after adjustments. The associations between ferritin and fatty acids or desaturases were no longer significant after adjusting for BMI, age and hs-CRP.

## 4. Discussion

Our study examined the association between iron and fatty acid metabolism in the development of impaired glucose tolerance in women with prior GDM. We found that women in the IGM group had numerous associations of SFAs, MUFAs and desaturases with iron, sTfR, TSAT and TIBC, whereas normoglycemic women showed few associations between fatty acids and iron parameters. Correlations were adjusted for BMI, age and hs-CRP, in order to control potential inflammation. Of particular note, sTfR was higher in the IGM group and associated positively with SFAs but negatively with MUFAs and desaturases, thus converting SFAs to MUFAs.

### 4.1. Differences between Glucose Tolerance Groups

As expected, the NGT group and IGM groups differed significantly when comparing anthropometrics, glucose and insulin status showing, as is widely recognized, that these factors are strongly connected to glucose tolerance [1].

When comparing iron status, the only differences found were lower serum iron, higher sTfR and lower TSAT in the IGM group. Previous studies have shown that increased levels of sTfR were connected to the development of T2D among an overweight and obese population, which may indicate that the connection between sTfR levels and T2D development is driven by obesity itself rather than by changes in iron metabolism [24,25]. However, a large study from 2012 did not show any correlation between sTfR and an increased risk for T2D [26]. It has also been shown that improving insulin sensitivity through exercise in obese women decreases sTfR [27]. Our study is the first to investigate these relationships in women previously diagnosed with GDM, and participants who are considerably younger compared to previous studies. The mean BMI in both groups were within the overweight interval (25–30 kg/m^2^) showing that, in women with previous GDM, a higher sTfR may be connected to insulin resistance, independent of obesity. When studying pregnant women there is also an urgent interest in the child and its future health. The first 1000 days of life, from conception to the second birthday, are widely accepted as important for the child’s future health and well-being. The impact on the child’s iron metabolism in women with GDM has been studied by Slomka et al. [28]. When studying iron parameters in serum cord blood of infants delivered by GDM mothers, they found no difference in prohepcidin, ferritin or sTfR concentration compared to control infants. Our research group follows a separate cohort of GDM women and their children prospectively, and it will be of high interest to examine the iron metabolism of these women and the iron parameters of the infants.

Our results displayed higher levels of the SFAs palmitic acid and myristic acid as well as D6D and lower levels of linoleic acid in the IGM group, which conforms with earlier findings [29,30], and is discussed at length in our previous studies [11].

### 4.2. Associations between Iron Parameters and Fatty Acid Metabolism

The most noticeable result of the current study was that the interactions between iron parameters and fatty acids changed with glucose tolerance. In women with impaired glucose metabolism, after adjustments for BMI, age and hs-CRP, numerous associations were found between iron parameters (iron, sTfR, TSAT, TIBC) and SFAs, MUFAs, or desaturases converting SFAs to MUFAs. For normoglycemic women, after the same adjustments, we found a positive association between ferritin and the PUFA DPA, while hepcidin associated negatively with the MUFA oleic acid and D6D. Even if we cannot determine the causality, it is interesting to see that the interplay between iron parameters and fatty acids clearly changes with glucose tolerance. This has not previously been shown in human studies.

Usually in high-cellular iron conditions, the transferrin receptor is downregulated. When iron levels are high, the iron regulative proteins (IRPs) stop binding to the iron responsive elements (IREs) on transferrin receptor mRNA, de-stabilizing the mRNA, downregulating the production of the transferrin receptor and decreasing the cellular uptake of iron. Our data show that, in people with impaired glucose tolerance, sTfR levels correlate with the levels of palmitic acid and myristic acid, and that they correlate with the IGM group but not with the NGT group, suggesting that the transferrin receptor and these SFAs interact differently with each other at different levels of glucose tolerance. In 2015 Dongiovanni et al. observed that rats which were fed a high-fat diet, with a predominance of SFAs, had increased hepatic iron accumulation mediated through the transferrin receptor [31]. They also showed an increased IRP activity and IRP protein levels compared to rats fed with a normal diet, and suggested that FAs interfered with iron metabolism in hepatocytes by inducing IRP and the transferrin receptor, thereby bypassing the negative feedback regulation. In 2019, Cui et al. demonstrated that the transferrin receptor plays an important role in insulin resistance caused by palmitic acid [32]. In their study on human skeletal muscle cells, they found that palmitic acid induced cellular iron overload by upregulating both the transferrin receptor protein and mRNA levels, leading to insulin resistance and glucose intolerance. In 2021, Zhang et al. showed that adipocyte-specific transferrin receptor deficiency, achieved through genetic modification, protected mice from high-fat, diet-induced metabolic disorders by restricting lipid absorption from the intestine through modulation of vesicular transport in enterocytes [33]. The distinct molecular mechanism that explains the means by which this fat-gut cross-talk occurs is still to be identified and clinically validated. As mentioned earlier, palmitic acid is strongly associated with the development of T2D, but data on the connection between transferrin receptor and T2D were inconsistent. A previous study examining sTfR in obese and non-obese patients found that although sTfR was associated with an increased risk of T2D in obese people, the inverse applied to non-obese people, thus showing a greater risk for T2D with lower levels of sTfR [25]. Our results may indicate a similar connection since sTfR correlated positively with palmitic acid in our metabolically unhealthy IGM group while trending towards an inverse association in the NGT group (Table S1). However, our results displayed an inverse association between sTfR and the MUFAs vaccenic acid and oleic acid, which emphasises the value of analysing the associations with each fatty acid separately, as was performed in this study. Several studies suggest that, while palmitic acid contributes to cell damage in pancreatic β-cells through lipotoxicity, monounsaturates and, specifically, oleic acid having a protective effect [21,34], our data do not prove this method to be applicable for humans. Nevertheless, it is intriguing that SFAs and sTfR in our population correlate in a similar way in women with IGM. If MUFAs on the other hand are protective against β-cell damage, it would be interesting to investigate further whether this is mediated through down regulation of the transferrin receptor. We believe that future studies on human subjects should investigate the iron status influence on intestinal lipid absorption, distinguishing the effect of iron parameters on absorption of SFAs, MUFAs and PUFAs, respectively, in order to better understand the molecular mechanisms and find targets for therapeutic interventions.

### 4.3. Strengths and Limitations

The complexity of studying how dietary intake affects health and illness lies in the variability of food composition, with many different nutrients with potential antagonistic, as well as synergistic, effects. In this study with human subjects, we have included several iron parameters and lipid profiling to unveil associations with all serum fatty acids. To our knowledge, no similar study has been performed in humans before and those performed in animal models or cell cultures were limited to fewer iron and FA parameters. Studies in the field have generally used fasting glucose or clinical diagnoses of T2D as outcome measures, while in this study, which was a more sensitive test of glucose metabolism, OGTT has been used to evaluate insulin levels and glucose tolerance in relation to iron homeostasis and FA profile. Additionally, the study includes a single-sex cohort with a narrow age span with the advantage that many variables are already controlled for. However, the possibilities to apply the results to an extended population are limited. Due to selection bias, the women who chose not to attend the follow-up visit had significantly higher plasma glucose levels, as well as BMI, compared to all the women eligible to participate. We assume that women who are more interested in health and nutrition are more prone to participate in a health-oriented study. Iron parameters and lipid profile have only been measured at one time point in this cross-sectional study, making it difficult to determine causality. Data collection at additional time points would be desirable to evaluate the risk of further diabetes development. Also, genetic confounders could potentially affect the relationship between iron, FAs and glucose tolerance. We have further used sTfR in serum as a measure of iron status, while the membrane-bound transferrin receptor is the active component in iron metabolism. Serum sTfR generally represents a constant proportion of the total mass of the tissue transferrin receptor; however, the proportion increases in case of iron deficiency, which could potentially affect our associations [35].

## 5. Conclusions

Women with previous GDM are at a higher risk of developing T2D and would benefit from extended knowledge about underlying metabolic mechanisms in development of the disease. Results showed that sTfR was higher and associated positively with SFAs and negatively with MUFAs in women that had developed impaired glucose tolerance six years after pregnancy. In general, numerous associations were seen between iron parameters and fatty acids in the IGM group, while very few significant associations were found in normoglycemic women. Preclinical studies have connected the transferrin receptor and SFAs in the development of insulin resistance. Our data indicate that these associations could also be applicable in a human population with impaired glucose tolerance following GDM. We propose that interactions between iron parameters, emphasising the transferrin receptor and FA metabolism, might be important in the development of pathological glucose tolerance. This may be mediated through an adipocyte–enterocyte cross-talk, as shown in rodents, and future nutritional studies, on human subjects, should investigate this further.

## Figures and Tables

**Table 1:** Characteristics of women with prior GDM at follow-up visit 6 years after pregnancy.

		NGT			IGM		NGT vs. IGM
	n	Mean	SD	n	Mean	SD	*p*-Value
Age (years)	99	39.4	4.9	58	38.5	5.6	0.289
Body mass index (kg/m^2^)	99	26.7	4.9	58	29.6	5.3	0.001 *
Waist circumference (cm)	98	87.7	11.0	57	94.7	12.7	0.001 *
Hip circumference (cm)	98	103.4	10.4	57	107.4	10.8	0.021 *
s-Insulin fasting (mU/L)	96	8.13	4.46	57	12.91	6.52	<0.001 *
s-Insulin 120 min (mU/L)	84	38.38	26.41	30 *	79.07	56.54	<0.001 *
p-Glucose fasting (mM)	99	5.37	0.37	58	7.19	2.47	<0.001 *
p-Glucose 120 min (mM)	91	5.49	1.09	31 ^a^	8.58	2.32	<0.001 *
HOMA-IR	95	1.97	1.10	57	4.20	2.64	<0.001 *
hs-CRP (mg/L)	99	2.41	4.03	58	4.87	7.51	0.006 *

The women were placed in groups, normal glucose tolerance (NGT) and impaired glucose metabolism (IGM). Comparisons between groups were analysed with independent *t*-test. Number (n), indicating number of accurate values possible to collect for each parameter measured. Standard deviation (SD). HOMA-IR, homeostatic model assessment of insulin resistance; hs-CRP, high sensitivity C-reactive protein. * Indicates significant differences. ^a^ Oral glucose tolerance test not completed for some participants due to diabetes diagnosis or too high fasting p-Glucose.

**Table 2:** Iron status of women with GDM at follow-up visit 6 years after pregnancy.

		NGT			IGM		NGT vs. IGM
	n	Mean	SD	n	Mean	SD	*p*-Value
s-Ferritin (µg/L)	98	59.61	45.43	58	61.50	80.37	0.064
s-Transferrin (g/L)	97	2.80	0.42	57	2.87	0.44	0.330
s-Iron (µmol/L)	95	16.81	5.41	57	15.04	5.77	0.037 *
s-Transferrin receptor (mg/L)	98	3.29	1.15	58	3.87	1.68	0.023 *
Transferrin saturation	94	0.25	0.09	57	0.22	0.09	0.038 *
s-TIBC (µmol/L)	98	67.88	14.30	58	70.27	14.02	0.229
s-Hepcidin (ng/mL)	87	40.20	41.06	55	33.60	33.24	0.394

The women were placed in groups, normal glucose tolerance (NGT) and impaired glucose metabolism (IGM). Comparisons between groups were analysed with independent *t*-test. Number (n), indicating number of accurate values possible to collect for each parameter measured. Standard deviation (SD). S-TIBC, serum total iron binding capacity. * Indicates significant differences.

**Table 3:** Fatty acid (FA) and desaturase status of women with GDM at follow-up visit 6 years after pregnancy.

			NGT			IGM		NGT vs. IGM
		n	Mean	SD	n	Mean	SD	*p*-Value
*Saturated FAs.* %								
Myristic acid	(14:0)	96	0.60	0.28	57	0.71	0.28	0.007 *
Palmitic acid	(16:0)	96	23.46	2.24	57	24.35	2.08	0.001 *
Stearic acid	(18:0)	96	8.35	0.98	57	8.55	1.03	0.189
*MUFAs.* %								
Palmitoleic acid (POA)	POA (16:1n7)	96	1.44	0.53	57	1.68	0.75	0.068
Vaccenic acid	(18:1n7)	96	2.03	0.30	57	2.04	0.32	0.961
Oleic acid	(18:1n9)	96	22.46	2.55	57	23.16	3.17	0.116
*ω-3 PUFAs.* %								
α-Linolenic acid	ALA (18:3n3)	96	0.71	0.23	57	0.78	0.28	0.179
Eicosapentaenoic acid	EPA (20:5n3)	96	0.92	0.59	57	0.95	0.42	0.310
Docosapentaenoic acid	DPA (22:5n3)	96	0.40	0.09	57	0.44	0.11	0.039 *
Docosahexaenoic acid	DHA (22:6n3)	96	1.74	0.63	57	1.64	0.53	0.592
*ω-6 PUFAs.* %								
Linoleic acid	LA (18:2n6)	96	30.31	4.10	57	27.68	3.97	<0.001 *
γ-Linolenic acid	GLA (18:3n6)	96	0.30	0.16	57	0.38	0.14	<0.001 *
Dihomo-γ-linolenic acid	DGLA (20:3n6)	96	1.25	0.32	57	1.40	0.37	0.021 *
Arachidonic acid	AA (20:4n6)	96	6.03	1.27	57	6.23	1.24	0.323
*Desaturase activity*								
Delta-5 desaturase	D5D (20:4n6/20:3n6)	96	5.25	1.77	57	4.87	1.70	0.155
Delta-6 desaturase	D6D (20:3n6/18:2n6)	96	0.05	0.02	57	0.06	0.02	<0.001 *
Stearoyl-CoA desaturase	SCD (16:1n7/16:0)	96	0.06	0.01	57	0.07	0.03	0.153
Delta-9 desaturase	D9D (18:1n9/18:0)	96	2.73	0.48	57	2.76	0.57	0.621

The women were placed in groups, normal glucose tolerance (NGT) and impaired glucose metabolism (IGM). Comparisons between groups were analysed with independent *t*-test. Number (n), indicating number of accurate values possible to collect for each parameter measured. Standard deviation (SD). * Indicates significant differences.

**Table 4:** Associations between FAs, desaturases and iron status in the NGT group.

Dependent	Independent		Unadjusted	Adjusted
Docosapentaenoic acid (22:5n3)	Ferritin	β-coefficient	0.210 *	0.213 *
		*p*-Value	0.041	0.032
Docosahexaenoic acid (22:6n3)	Iron	β-coefficient	0.232 *	0.147
		*p*-Value	0.026	0.166
Docosahexaenoic acid (22:6n3)	Transferrin saturation	β-coefficient	0.221 *	0.131
		*p*-Value	0.035	0.218
Oleic acid (18:1n9)	Hepcidin	β-coefficient	−0.291 *	−0.275 *
		*p*-Value	0.007	0.010
Delta-6 desaturase (20:3n6/18:2n6)	Hepcidin	β-coefficient	−0.250 *	−0.228 *
		*p*-Value	0.022	0.032

Unadjusted and adjusted linear regression models. Adjustments made for BMI, age, and hs-CRP. * Indicates significant differences.

**Table 5:** Associations between FAs, desaturases and iron status in the IGM group.

Dependent	Independent		Unadjusted	Adjusted
Palmitoleic acid (16:1n7)	Ferritin	β-coefficient	0.298 *	0.228
		*p*-Value	0.024	0.070
Stearoyl-CoA desaturase (16:1n7/16:0)	Ferritin	β-coefficient	0.271 *	0.206
		*p*-Value	0.042	0.104
Palmitoleic acid (16:1n7)	Iron	β-coefficient	0.232	0.250 *
		*p*-Value	0.085	0.048
Vaccenic acid (18:1n7)	Iron	β-coefficient	0.319 *	0.333 *
		*p*-Value	0.017	0.014
Oleic acid (18:1n9)	Iron	β-coefficient	0.321 *	0.302 *
		*p*-Value	0.016	0.018
Stearoyl-CoA desaturase (16:1n7/16:0)	Iron	β-coefficient	0.299 *	0.313 *
		*p*-Value	0.025	0.013
Delta-9 desaturase (18:1n9/18:0)	Iron	β-coefficient	0.308 *	0.317 *
		*p*-Value	0.021	0.018
Myristic acid (14:0)	Transferrin receptor	β-coefficient	0.284 *	0.274 *
		*p*-Value	0.033	0.039
Palmitic acid (16:0)	Transferrin receptor	β-coefficient	0.305 *	0.273 *
		*p*-Value	0.021	0.034
Vaccenic acid (18:1n7)	Transferrin receptor	β-coefficient	−0.248	−0.265 *
		*p*-Value	0.063	0.048
Oleic acid (18:1n9)	Transferrin receptor	β-coefficient	−0.236	−0.251 *
		*p*-Value	0.077	0.046
Vaccenic acid (18:1n7)	Transferrin saturation	β-coefficient	0.303 *	0.322 *
		*p*-Value	0.023	0.018
Oleic acid (18:1n9)	Transferrin saturation	β-coefficient	0.283 *	0.281 *
		*p*-Value	0.035	0.027
Stearoyl-CoA desaturase (16:1n7/16:0)	Transferrin saturation	β-coefficient	0.252	0.285 *
		*p*-Value	0.061	0.024
Delta-9 desaturase (18:1n9/18:0)	Transferrin saturation	β-coefficient	0.299 *	0.322 *
		*p*-Value	0.025	0.016
Stearic acid (18:0)	TIBC	β-coefficient	0.257	0.276 *
		*p*-Value	0.054	0.043

Unadjusted and adjusted linear regression models. Significant correlations displayed in bold. Adjustments made for BMI, age, and hs-CRP. * Indicates significant differences.

## Data Availability

The data presented in this study are available on request from the corresponding author. The data are not publicly available due to data protection regulations.

## Supplementary Materials

The following supporting information can be downloaded at: https://www.mdpi.com/article/10.3390/nu15143214/s1, Table S1: Associations between FAs, desaturases and iron status in the NGT group, Table S2: Associations between FAs, desaturases and iron status in the IGM group.
